# Essential redundancies fuel *Mycobacterium tuberculosis* adaptation to the host

**DOI:** 10.1371/journal.ppat.1013749

**Published:** 2025-12-05

**Authors:** Marco Silva, Alexandre J. Pinto, Tiago Beites

**Affiliations:** 1 i3S—Instituto de Investigação e Inovação em Saúde, University of Porto, Porto, Portugal; 2 Doctoral Program in Molecular and Cell Biology, ICBAS—Instituto de Ciências Biomédicas Abel Salazar, University of Porto, Porto, Portugal; University of Queensland, AUSTRALIA

## Abstract

Redundancy in biology is, at a glance, counterintuitive because if the function of two gene products completely overlaps then, throughout the course of evolution, one of the genes will likely accumulate mutations to the point of loss-of-function. The consensus is that partial functional overlap, for example, divergent secondary functions, play a major role in redundancy conservation. This asymmetrical nature offers a crucial advantage: phenotypic plasticity, which ensures that an essential cellular function can adapt to changes in the environment. In this context, the human pathogen *Mycobacterium tuberculosis* is an interesting example. Despite being an obligate pathogen that has been co-evolving with the human host for millennia, *M. tuberculosis* genome retains redundant functions at multiple levels that allow the bacilli to adapt to extremely heterogeneous environments in the human host. This review explores how *M. tuberculosis* functional redundancies mirror the heterogeneity of both intra- and extracellular host niches, with a focus on energy metabolism. Finally, we discuss the challenges and opportunities of functional redundancies in the context of drug development.

## 1. Introduction

Redundancy can be defined as an excess of causal factors beyond the minimum required for a given function. In biology, complete functional redundancy should not result in an evolutionarily stable solution, because one of the genes involved should randomly accumulate mutations possibly culminating in loss-of-function. So, how did redundancy become conserved? James H Thomas proposed four types of selective processes for the conservation of redundancy in genomes [1]. Selection by cumulative function, where one copy of a given gene does not render enough product to sustain function, thus needing multiple copies. Selection by increased fidelity, where multiple genes are required for maximum fidelity in a process. Selection by divergent functions, which is observed when two gene products have overlapping functions but are under selection for divergent functions. Selection by emergent properties, where two gene products have a similar function and, when present at the same cell compartment, lead to an emergent property that increases fitness. In all cases, a functional asymmetry seems to be necessary for redundancy to be stable in the long term [2].

In living organisms, functional redundancy can occur at multiple scales [3]. Genetic redundancy refers to gene products within a given genome that perform the same biochemical function. Classically, genetic redundancy is equated with gene duplication events, but it is also the case that phylogenetically unrelated genes can provide the same function through analogous evolution [2]. Above the single gene level, redundancy can also occur between unrelated metabolic pathways/cell processes. This is observed, for example, in microorganisms that harbor the capacity to obtain a given metabolite through a dedicated biosynthetic pathway or through a salvage pathway [4]. Above the organism level, one can also find genotypic redundancy, which refers to different genotypes in a population that can achieve the same phenotype [5].

Essential gene is an important concept in functional redundancy and can be defined as encoding a gene product that performs a biochemical function essential for growth and/or survival and for which there are no compensatory mechanisms [6]. In bacterial functional biology, essentiality calls are based on genetic screens like transposon-sequencing [7] or CRISPR interference [8] that target the whole genome. Of note, a subset of these genes is only essential in certain environments and are thus classified as conditionally essential [9]. In bacterial pathogens, genetic screens during infection identified conditionally essential genes *in vivo* that are dispensable for *in vitro* bacterial growth [10]. Interestingly, the expansion of bacterial functional screens to the infection of a genetically diverse set of mice clearly showed that the subset of pathogen conditionally essential genes is also highly dependent on the host genetic makeup [11]. Genes essential for growth and/or survival in a given environment perform essential functions, but not all essential functions are carried out by essential genes, raising the question: why do some essential functions encompass extensive genetic redundancy while others are based on essential genes? One possible answer is that some cellular processes require different components to remain functional when the environment changes [12]. The asymmetric nature of functional redundancy is a source of phenotypic plasticity and, thus, provides a solution to this adaptive problem.

In this context, the human pathogen *Mycobacterium tuberculosis*—tuberculosis (TB) etiological agent—is an interesting example. Although it has been co-evolving with humans for millennia, it maintains a high degree of functional redundancy encoded in its genome [13]. This contrasts, for example, with *Mycobacterium leprae*—leprosy etiological agent—a human pathogen that followed a genome reduction adaptive strategy with less discernible redundant systems [14]. *M. tuberculosis* successfully infects different host cell types and even thrives in extracellular environments like caseum (necrotic center of caseating granulomas) [15,16]. In turn, although *M. leprae* can be found in other host cells, it is a specialist predominantly adapted to infect Schwann cells of the peripheral nervous system [17]. These infection strategies are thus mirrored in the level of functional redundancy encoded in the genomes of both pathogens. Beyond mycobacteria, obligate human pathogens typically display minimized and/or streamlined genomes with low functional redundancy. *Treponema pallidum* (syphilis) [18], *Chlamydia trachomatis* (chlamydial infections) [19], *Mycoplasma pneumoniae* (lung infection) [20], and *Mycoplasma genitalium* (genital infection) [21] followed a genome reduction evolutionary path. *Bordetella pertussis* (whooping cough) adaptation to the human host led to moderate genome reduction; however, *B. pertussis* is characterized by extensive gene loss-of-function through propagation of insertion sequences (IS), minimizing functional redundancy [22]. *Neisseria gonorrhoeae* (gonorrhoea) and *Neisseria meningitidis* (meningitis) also present moderate genome reduction with redundancy mainly restricted to virulence genes [23]. *M. tuberculosis* is, thus, an outlier among obligate human pathogens. Indeed, co-evolution with the human host sculpted a metabolic network that in many nodes departs from canonical pathways either by functional redundancies or by divergent ways of operating (for a review discussing *M. tuberculosis* metabolic evolution with illustrative examples see [24]; for a general review on *M. tuberculosis* evolution see [25]).

In this review, we discuss *M. tuberculosis* energy metabolism as a paradigmatic case of functional redundancies that ensure adaptation to heterogeneous niches encountered in the host during infection. We begin by outlining the varied host environments *M. tuberculosis* encounters, followed by an analysis of how redundancies enable the bacilli to meet these challenges. Finally, we discuss the lessons learned from exploring *M. tuberculosis* biological redundancies to TB drug development.

## 2. A heterogeneous host environmental landscape

*M. tuberculosis* host niches of infection are heterogeneous and dynamic. Even within the same individual, TB lesions can follow dramatically different paths, from total resolution to the formation of cavitary lesions [26]. *M. tuberculosis* infection can be divided into intracellular and extracellular niches [15,16]. This entails that *M. tuberculosis* had to develop enough phenotypic plasticity to thrive in such different environments.

### 2.1. Intracellular niches for *M. tuberculosis*

In the early instances of *M. tuberculosis* infection, the bacilli are primarily phagocytosed by myeloid cells, such as macrophages, dendritic cells, and neutrophils [27–29]. Less frequently, *M. tuberculosis* bacilli can also be found in non-myelocytic cells, such as type-I and -II epithelial cells [15,30–32]. Considering the lung myeloid cell landscape, the intracellular conditions for *M. tuberculosis* growth tend to vary. Regarding macrophages, the primary intracellular habitat, two ontologically distinct lineages of macrophages have been described: alveolar and interstitial macrophages [33]. Alveolar macrophages, which first come in contact with the pathogen [34], display upregulated fatty acid (FA) uptake and β-oxidation, and provide a more permissive environment for bacterial growth. Interstitial macrophages, on the other hand, undergo a glycolytic shift that ultimately deprives *M. tuberculosis* of nutrients and control bacterial growth [33]. This metabolic switch to aerobic glycolysis has several repercussions in the intracellular microenvironment [35]. Glycolytic fluxes lead to pyruvate synthesis, which is mainly used as a precursor for lactate and/or alanine synthesis. This overall increase in total carbohydrate content of the infected cells aims to refuel key pathways for bacterial control. This is the case of glycolysis and the pentose phosphate pathway, which supply ATP, NADH, and biosynthetic precursors to antimicrobial responses like phagocytosis, reactive oxygen species (ROS) and nitric oxide production, and cytokine release [36,37]. Neutrophils also constitute an early niche environment for *M. tuberculosis* [27]. Although capable of microbicidal activity, they can provide a permissive environment and their accumulation correlates with tissue damage and disease severity [38–40]. Recent studies highlight neutrophil increased FA metabolism as a key factor for the permissiveness of these cells, acting as FA and cholesterol reservoirs for *M. tuberculosis* [39,41].

As disease progresses, infected cells disseminate into the lung interstitium, where local inflammation and lymphocyte influx from draining lymph nodes drive the formation of granulomas—organized multicellular aggregates with infected macrophages at the core encircled by a lymphocytic cuff [42,43]. This environment both restricts the growth and promotes survival of *M. tuberculosis* in a slow to non-replicative state [44,45]. Granulomas are not homogeneous and different structures may co-exist in the same individual [26,46], resulting in variable levels of nutrient availability [47]. The oxygen tension in granulomas is low (1.61 ± 0.31 mmHg) [48], which is probably maintained within this range due to constant oxygen diffusion [49]. Despite being an obligate aerobe that requires oxygen to grow, *M. tuberculosis* can survive hypoxic conditions through a series of metabolic adaptations [50]. Hypoxic, infected macrophages in granulomas become “foamy” with abundant lipid droplet (LD) accumulation [51–53]. Recent work shows this LD formation is largely a host immune response rather than a manipulation strategy initiated by *M. tuberculosis*. Knight and colleagues found that IFN-γ/HIF-1α-mediated signaling induces LD formation as part of a host defense mechanism [54]. Indeed, IFN-γ induced LDs actually impeded bacterial access to host lipids, and contributed to the production of host-protective lipid mediators (e.g., PGE_2_ and LXB_4_) that limit bacterial survival [54]. In other words, LD biogenesis here acts as a lipid “sink” in the host, sequestering FA away from the pathogen’s reach. In contrast, *M. tuberculosis* can directly exploit host lipids independently of LD. Daniel and colleagues demonstrated that in hypoxic, lipid-loaded macrophages the bacilli import FA from host triacylglycerol (TAG) and incorporate them into their own TAG inclusions [55]. Consistent with this, transcriptomic profiling of *M. tuberculosis* in infected adipocytes (a lipid-rich model) revealed downregulation of bacterial *de novo* FA synthesis and upregulation of triglyceride biosynthesis genes [56]. These changes suggest that the bacilli rely on host FA rather than synthesizing them. Moreover, infection alters LD composition, as shown by quantitative proteomics of macrophage LD during *M. tuberculosis* infection [57]. Menon and colleagues observed increased abundance of LD-associated proteins involved in lipid metabolism, protein synthesis, and vesicular trafficking, suggesting the bacterium actively alters host LD, exploiting their content for growth [57]. This further shows that although LD formation is host-initiated, the pathogen can remodel LD proteomes after their formation, perhaps to access or repurpose LD content. Importantly, the host’s overall lipid processing determines bacterial nutrient availability. Simwela and colleagues demonstrated that genetically or pharmacologically blocking macrophage FA uptake, storage or catabolism severely impacts intracellular *M. tuberculosis* growth and elicits multiple antimicrobial responses [58]. Macrophages unable to import or store FA show increased glycolysis, oxidative stress (ROS production), pro-inflammatory cytokines and autophagy, all of which suppress *M. tuberculosis* replication [58]. Essentially, *M. tuberculosis* thrives only when it can exploit host lipids in macrophages with high lipid uptake, synthesis and turnover, whereas LD formation itself represents an IFN-γ-driven lipid-sequestration defense [54,58].

### 2.2. Extracellular niches

In advanced granulomas, infected foamy macrophages in the center become necrotic, releasing their lipid-rich content and bacteria to the extracellular environment, forming the caseum [59]. Studies revealed that the protein and lipid composition of caseum resembled the intracellular environment of foamy macrophages [60,61]. However, unlike the intracellular setting, caseum represents an environment of sustained nutrient access. This lipophilic environment induces accumulation of lipids inside the bacilli, in the form of intracellular lipophilic inclusions (ILI). Studies showed that the major lipid species found in lipid extracts from ILI-rich *M. tuberculosis* include TAG, FA, polar lipids, and wax esters [55,62,63], reflecting the lipid availability of caseum [16]. Low oxygen tension is also a well-known feature of caseous granulomas, given the lack of vascularization in the granuloma core [64]. This is further enhanced by the fibrotic cuff that surrounds advanced granulomas [16]. The mean oxygen partial pressure of caseous granulomas was 1.6 mmHg, 37-fold lower than that of seemingly normal sections of lung [65]. In more advanced stages, the granuloma may rupture, leading to cavitary disease [59]. These cases are associated with active transmission, as granulomas rupture at the alveoli and come in contact with the airway. Here, the oxygen tension that the bacilli face is similar to that of bronchi [65], promoting resumption of replication and consequently to higher bacterial burden.

*M. tuberculosis* co-evolution with humans has resulted in a wide variety of host environments and in a remarkable phenotypic plasticity in the pathogen.

## 3. Redundancies in *M. tuberculosis* energy metabolism as an adaptation to host heterogeneous environments

During infection, *M. tuberculosis* utilizes lipids, namely cholesterol and FA, as preferred carbon sources [66]. Cholesterol is catabolized via enzymatic degradation of the rings structure and β-oxidation of the side chain, while FA are solely degraded via β-oxidation (reviewed in [67]). Although available in environments like caseum, glycolytic carbon sources are dispensable for growth and survival during infection [16,68,69]. Being an obligate aerobe, lipid uptake and catabolism need to be coordinated with the respiratory chain to obtain energy, which require a significant phenotypic plasticity. In this section, we will review the extensive functional redundancies in *M. tuberculosis* energy metabolism (Table 1) and discuss their contribution to host adaptation.

**Table 1:** Functionally redundant enzymes in *Mycobacterium tuberculosis* energy metabolism.

Gene name	Enzyme name	Enzymatic activity
**Energy sources**		
*fadD1-D19, fadD34-D36*	Fatty acyl-CoA ligase (FACL)	FACL activates fatty acids with a CoA molecule and determines their metabolic fate to the catabolic route
*fadE1-E10, fadE12-E13, fadE15-E36*	Acyl-CoA dehydrogenase (ACAD)	Generates a trans-double bond between the α and β carbons of the acyl-CoA substrate
*echA1-A21*	Enoyl-CoA hydratase (ECH)/ Enoyl-CoA isomerase (ECI)	ECH catalyzes the hydroxylation of the carbon chain at the β position. In the case of unsaturated fatty acids, a step of isomerization catalyzed by ECI occurs before the ECH-catalyzed step
*fadB2-B5*	Hydroxyacyl-CoA dehydrogenase (HAD)	Introduces a keto group at the β position
*fadA2-A6*	Thiolase (TH)	Cleaves acyl-CoA chain and releases an acetyl-CoA molecule
*fadB; fadA*	Trifunctional enzyme complex (TFE)	ECH, HAD, and TH activities
*icl1; icl2*	Isocitrate lyase	ICL catalyzes a Mg² ⁺ -dependent cleavage of isocitrate (glyoxylate cycle) or methylisocitrate (methylcitrate cycle)
**Energy generation**		
*nuoA-J* (operon)	NADH dehydrogenase type-I (Ndh-1)	Oxidizes NADH and reduces menaquinone; proton-pumping activity
*ndh; ndhA*	NADH dehydrogenase type-II (Ndh-2)	Oxidizes NADH and reduces menaquinone
*Rv0247c-Rv0249c* (Sdh1 operon); *sdhCDAB* (Sdh 2 operon)	Succinate dehydrogenase	Oxidizes succinate to fumarate and reduce menaquinone
*ctaB; ctaC; ctaD;* *ctaE-qcrCAB* (operon)	Cytochrome *bc1*-*aa3* oxidase	Oxidizes menaquinone and transfers the electrons to molecular oxygen (last electron acceptor); proton-pumping activity
*cydAB*	Cytochrome *bd* oxidase	Oxidizes menaquinone and transfers the electrons to molecular oxygen (last electron acceptor)

### 3.1. Energy sources

**Fig 1:**
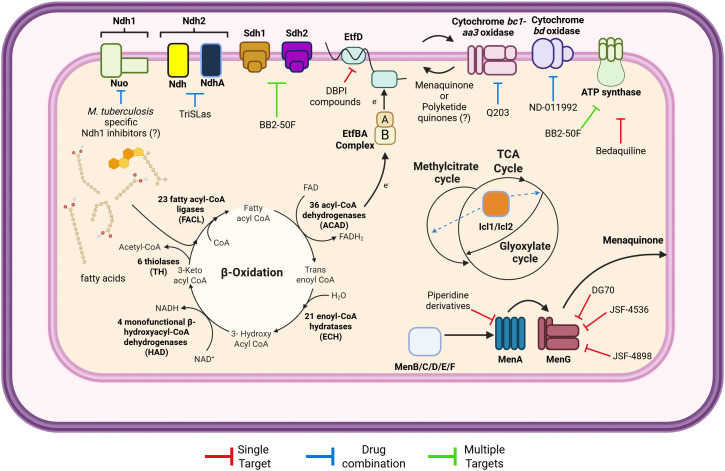
Overview of functional redundancies and inhibitors of *Mycobacterium tuberculosis* energy metabolism. Energy metabolism in *M. tuberculosis* is notorious for functional redundancies. β-oxidation has multiple paralogs for each enzymatic step, which can be both an adaptation to the need to detoxify fatty acids, and the need to optimize their use as carbon sources. In this pathway, only EtfD/EtfBA complex, which is necessary for the activity of multiple ACAD, is essential *in vitro* and *in vivo*. Icl1 and Icl2 participate in both the glyoxylate cycle and methylcitrate cycle. Regarding oxidative phosphorylation, ATP synthase was shown to be essential in vitro and for infection, while menaquinone biosynthesis pathway was shown to be essential in vitro – a possible redundancy with polyketide quinones remain to be tested. The remaining components show a remarkable degree of functional redundancy, with NADH dehydrogenases and terminal oxidoreductases being the most well-studied cases. *M. tuberculosis* targeting energy metabolism follows three main modalities: (i) inhibition of essential enzymes—single target; (ii) drug combination, where treatment with two compounds overcomes functional redundancy; (iii) compounds that inhibit multiple targets. Created with BioRender.com.

Functional genomics and follow-up genetics studies revealed that cholesterol degradation genes are essential for cholesterol utilization and full virulence [70–73]. In contrast, FA β-oxidation-related genes are dispensable for infection [10], and none was shown to be essential for the degradation of FA (Fig 1). This non-essentiality is the result of extensive functional redundancy, with multiple paralogs for each β-oxidation step [13]: 22 putative fatty acyl-Coenzyme A (CoA) ligases (FACL); 35 putative acyl-CoA dehydrogenases (ACAD); 21 putative enoyl-CoA hydratases (ECH); 4 putative monofunctional β-hydroxyacyl-CoA dehydrogenases (HAD); and 6 putative thiolases (TH). Hence, what selective pressure(s) drove the conservation of multiple genes encoding enzymes to perform the same function? Two non-mutually exclusive selective pressures may have played a role in the conservation of β-oxidation paralogs: (i) the need to optimize FA catabolism to maximize growth and/or avoid FA bactericidal effects [74,75] may have led to selection by cumulative function; (ii) FA found in infectious foci vary in chain length, ramification and degree of unsaturation [69,76], which may have led to selection by divergent function to cover FA structural diversity. Next, we will delve on what is known about *M. tuberculosis* FA β-oxidation enzymes and their contributions to infection.

Biochemical characterization of *M. tuberculosis* FACL revealed a wide functional overlap. FadD2 uses palmitic acid as a preferred substrate but also shows activity with myristic and oleic acids [77]. FadD5 is implicated in the degradation of mycolic acids but not in linear FA, hinting for a role in cell wall recycling [78]. Interestingly, a *fadD5* deletion mutant shows attenuated growth in a mouse infection model, which may be due to defective cell wall recycling [78]. FadD8 shows preference for medium-chain FA, although it is likely to also take part in the degradation of cholesterol side-chains [79,80]. FadD13 has been described as a very-long-chain FA acyl-CoA ligase, although it can also bind to long-chain FA, such as palmitic acid [81,82]. Biochemical characterization of FACL, thus, demonstrates that *M. tuberculosis* can activate a wide range of FA for downstream degradation using enzymes with overlapping but divergent substrate affinities.

Within the group of ACAD involved in FA β-oxidation, only FadE5 is well-characterized biochemically. This enzyme shows a broad affinity to linear fatty acyl-CoA (4–22 carbon atoms) [83], but it is not predicted to be essential during infection [10]. Although this enzyme shows a wide substrate affinity, it does not cover, for example, branched FA, which is suggestive of functional redundancy. In fact, only the inactivation of multiple ACAD is capable of blocking β-oxidation and abrogate growth and survival during infection [74].

ECH enzymes in *M. tuberculosis* were first predicted to display bifunctional enzymatic activity [13]: ECH and enoyl-CoA isomerase (ECI). Interestingly, it has been shown that many *echA* genes encode for monofunctional ECI (EchA2/A5/A10/A11/A16), while others demonstrate bifunctional activity (EchA1/A8/A9) [84]. Regarding substrate specificity, all these enzymes were able to react with 3-cis octenoyl-CoA. However, other ECH tested were not reactive with this substrate (EchA4/A6/A7/A14/A15/A17/A19/A20/A21) [84], suggesting that selection by divergent function played a role in *M. tuberculosis* ECH evolution.

The last steps of FA β-oxidation are catalyzed by HAD and TH. From the predicted monofunctional HAD in *M. tuberculosis*, only FadB2 enzymatic activity was experimentally confirmed (β-hydroxybutyryl-CoA as substrate) [85,86]. Regarding TH, based on structure homology, FadA2 was predicted to have substrate specificity for long-chain FA [87]. Further studies are needed to understand if substrate affinity varies among HAD and TH monofunctional enzymes.

Like other bacteria, FA β-oxidation in *M. tuberculosis* accounts with a trifunctional enzyme complex (TFE), composed of FadB (ECH and HAD activities) and FadA (TH). The experimentally demonstrated absence of ECI activity in the TFE complex makes it inactive against unsaturated FA [84]. Characterization of substrate specificity of the TFE complex showed higher affinity for medium- to long-chain FA [84]. TFE complex was not demonstrated to be essential for the utilization of FA and it is not predicted to be essential during infection [10], strongly suggesting the presence of redundant enzymes.

Other notable example of functional redundancy in pathways associated with carbon catabolism in *M. tuberculosis* is isocitrate lyase (bifunctional: glyoxylate cycle and methylcitrate cycle), which accounts with two paralogs Icl1 and Icl2 [88,89]. Deletion of *icl1* alone impairs growth on propionate and mildly impacts chronic phase survival in mouse lungs, while deletion of *icl2* has apparently no physiological effect [88,90]. However, double deletion of *icl1* and *icl2* abrogates the ability to grow on lipids as carbon sources and leads to rapid eradication of infection in mouse lungs [88]. This shows the characteristic functional asymmetry among redundant enzymes, with Icl1 being more relevant for the catabolism of propionate-generating carbon sources like cholesterol and odd-chain fatty acids. Interestingly, isocitrate lyases were also shown to be necessary for the utilization of lactate and pyruvate, possible carbon sources inside macrophages [91,92].

Whether due to the need of a high quantity of enzyme or the need to accommodate diverse structures, *M. tuberculosis* is equipped with phenotypic plasticity to take advantage of a wide variety of carbon sources to grow and survive in different host niches.

### 3.2. Energy generation

*M. tuberculosis* oxidative phosphorylation is essential to meet its energetic needs [93]. Operationally, the electron transport chain that supports oxidative phosphorylation can be divided in 1) enzymes that transfer electrons to the respiratory chain, 2) electron carrier molecules, and 3) terminal oxidoreductases that transfer electrons to the final acceptor (reviewed in [94]) (Fig 1). Throughout the movement of electrons across the different components, a proton motive force (PMF) is generated, which drives the generation of ATP through the activity of ATP synthase. *M. tuberculosis* oxidative phosphorylation encompasses several functional redundancies, with menaquinone biosynthesis and ATP synthase being the only enzymes essential for growth *in vitro* [7,8,95]. In this subsection, we will describe functional redundancies on *M. tuberculosis* respiratory chain enzymes and discuss how they contribute to adaptation to the host.

*M. tuberculosis* expresses two enzymes with NADH dehydrogenase activity: type-1 NADH dehydrogenase (Ndh-1) that is homologous to the human respiratory complex I and type-2 NADH dehydrogenases (Ndh-2). There are multiples levels of functional redundancy in *M. tuberculosis* NADH dehydrogenases. At the level of gene duplication, *M. tuberculosis* expresses two paralogs of Ndh-2: Ndh and NdhA [13]. According to functional genomics, *ndh* was classified as associated with growth defect, while *ndhA* was classified as non-essential [7,8,95]. Complementation of a *ndh*/*ndhA* double knockout mutant with only *ndh* expressed constitutively does not completely rescue the loss of viability in hypoxia and optimal growth in mice lungs [75]. Moreover, evaluation of mice survival revealed that a *M. tuberculosis* knockout strain for *ndh* is attenuated, whereas the knockout strain for *ndhA* was as virulent as the parental strain [96]. This phenotypic asymmetry could be due to different expression patterns depending on environmental cues, secondary catalytic activities, or even selection by cumulative function. Nevertheless, the conservation of Ndh and NdhA in *M. tuberculosis* is a largely unanswered question. A second level of redundancy occurs between Ndh-1 and Ndh-2 through analogous function. In certain environments, like axenic medium devoid of FA, *M. tuberculosis* knockout strain for *ndh* and *ndhA* (Δ*ndh-2*) behaves as the wild-type [75]. Additionally, in these conditions, chemical inhibition of Ndh-1 in the Δ*ndh-2* strain leads to bacterial death [75,97]. *In vivo*, a Δ*ndh-2* mutant presents an attenuated growth in mice [75], while a Δ*ndh*Δ*nuoAN* mutant has a survival defect [96]. Hence, the available evidence shows that Ndh-1, at least in some contexts, can compensate for the lack of a functional Ndh-2. However, in conditions that shift the redox balance to a reduced state, such as the consumption of highly reduced carbon sources (e.g. long-chain FA) or hypoxia, Ndh-2 becomes essential [75]. Ndh-2 allows for a higher turnover of NADH into NAD+, thus contributing more efficiently to the maintenance of the function of important enzymes (e.g. TCA cycle enzymes) and to intracellular redox homeostasis [75]. Moreover, its non-proton-pumping nature makes it more resistant to backpressure when PMF is high. In turn, due to its proton-pumping activity, Ndh-1 may be important to provide enough energy in environments where *M. tuberculosis* is actively dividing. The partial functional overlap and cumulative effect between Ndh-1 and Ndh-2 is a very successful adaptive mechanism that allows *M. tuberculosis* to survive non-replicative states and maximize growth rates when nutrients and oxygen are available. A third layer of redundancy is observed at the level of electron input to the respiratory chain. Heterologous expression of a NADH oxidase (Nox), which oxidizes NADH to NAD+, rescued *M. tuberculosis* Δ*ndh-2* from the bactericidal activity of Ndh-1 inhibitors [97]. Although this is an artificial system, it shows that *M. tuberculosis* has the potential to rely on alternative electron donors to meet its energetic needs. Enzymes such as succinate dehydrogenase or malate:menaquinone oxidoreductase [94] are good candidates to play this compensatory role on electron input.

Succinate dehydrogenases play an important role in energy metabolism by coordinating central carbon metabolism (TCA cycle) and respiration [98]. *M. tuberculosis* expresses three enzymes capable of oxidizing succinate to fumarate: succinate dehydrogenase 1 (Sdh1), succinate dehydrogenase 2 (Sdh2) and fumarate reductase (possibly catalyzing the reaction bidirectionally) [94,99]. Simultaneous silencing of Sdh-1 and Sdh-2 operons abrogates succinate oxidation with a concomitant impairment in growth [99]. In C3HeB/FeJ mice, a *M. tuberculosis* strain defective for Sdh1 presented an attenuated phenotype in lungs, while the absence of Sdh2 did not impact final bacterial loads [98]. This suggests that the functional redundancy is only partial, and thus the presence of two operons encoding functional succinate dehydrogenases may be the result of selection by cumulative and/or divergent function.

Electron carriers are hinge molecules that get reduced by enzymes that donate electrons to the respiratory chain and mediate electron transport to terminal oxidoreductases. In *M. tuberculosis*, the main electron carrier is menaquinone [94]. Genes encoding for menaquinone biosynthetic enzymes are essential for *in vitro* growth across different functional genomics datasets [7,8,95]. Accordingly, inhibitors of menaquinone biosynthesis enzymes, namely MenA (e.g. piperidine derivatives) [100,101], or MenG (e.g. DG70, JSF-4536 and JSF-4898) [102,103] showed bactericidal activity against *M. tuberculosis in vitro*. The essentiality of menaquinone biosynthesis during infection remains a largely unanswered question. In a recent study, the MenG inhibitors JSF-4536 and JSF-4898 did not show activity against *M. tuberculosis* in BALB/c mice individually [103]. The authors point out that the lack of activity *in vivo* might be a result of the chemical nature of the compounds rather than the possible non-essentiality of menaquinone biosynthesis. However, the picture may be more complex, given that it has been reported that *M. tuberculosis* can synthesize a possible alternative electron carrier [104]. This means that there may be functional redundancy at the pathway level. In *Mycobacterium smegmatis* growing in biofilms, polyketide quinones were shown to constitute an alternative electron carrier with special importance in hypoxic microenvironments [104]. The presence of polyketide quinones in *M. tuberculosis* membranes [104], together with a report supporting the importance of biofilm formation for infection [105], suggest the possibility of an electron carrier functional redundancy at least linked to oxygen availability—a hypothesis that merits further testing.

*M. tuberculosis* expresses two phylogenetically unrelated terminal oxidoreductases capable of oxidizing menaquinone and transferring the electrons to molecular oxygen as the last acceptor—cytochrome *bc1*-*aa3* and cytochrome *bd* oxidase [13]. Cytochrome *bc1*-*aa3* oxidase is a multiprotein complex composed of the *bc1*-complex (encoded by *qcrCAB*) and an *aa3*-type cytochrome c oxidase (encoded by *ctaBCDE*). Cytochrome *bd* oxidase in an enzyme encoded by the genes *cydAB.* Genetic inactivation or chemical inhibition of cytochrome *bc1-aa3* leads to a bacteriostatic effect *in vitro* and *in vivo*, while inactivation of cytochrome *bd* oxidase has no apparent effect on *M. tuberculosis* growth and survival *in vitro* or *in vivo* [75,106,107]. However, when both enzymes are simultaneously inactivated, either genetically or chemically, a bactericidal effect is observed *in vitro* and *in vivo* [75,107,108]. As with other functional redundancies, there is also a functional asymmetry that may explain the conservation of both enzymes in *M. tuberculosis*. Because cytochrome *bd* oxidase has high affinity to oxygen [109] and its expression is induced under hypoxic conditions [110], it has been hypothesized that this enzyme could be essential in environments where oxygen is scarce [94]. However, a genetic screen in hypoxia did not identify cytochrome *bd* oxidase as essential [50]. Also, it has been shown that cytochrome *bc1*-*aa3* oxidase is functional even at very low oxygen concentrations (0.02 mmHg) [111]. Both sets of evidence do not support cytochrome *bd* oxidase as an adaptive mechanism to variations in oxygen availability. Surprisingly, cytochrome *bd* oxidase was shown to contribute for survival in IFN-γ-activated macrophages [112]. In specific, *M. tuberculosis* relies on cytochrome *bd* oxidase for respiration in acidic environments [112]. This suggests that cytochrome *bd* oxidase can be an adaptive strategy to changes in the environmental pH.

In all, the documented functional redundancies in *M. tuberculosis* oxidative phosphorylation provide enough phenotypic plasticity for this pathogen to adapt to environments with different combinations of carbon sources, oxygen availability and environmental pH. If we take the example of the environmental conditions faced by *M. tuberculosis* within granulomas [16,44,45], one can conclude that a highly redundant energy metabolism is key for a successful infectious cycle.

## 4. Targeting functional redundancies

Given the need to develop shorter, better tolerated and efficient TB treatment regimens, in this section [113], we will discuss opportunities and strategies to target redundancy (Fig 1).

A “low hanging fruit” strategy is to find essential enzymes within a highly redundant cellular process. In oxidative phosphorylation, the most well-known example in *M. tuberculosis* is ATP synthase – the target of bedaquiline, a drug that revolutionized the treatment of drug-resistant TB [114,115]. Inhibition of menaquinone biosynthetic enzymes could, in principle, also constitute a strategy to kill *M. tuberculosis* during infection. However, the essentiality of this pathway, and activity of inhibitors *in vivo* remains to be demonstrated [103]. Another possibility could be EtfD inhibition, which is required for the activity of multiple ACADs and, thus, essential for a functional β-oxidation [75]. In fact, although an auxiliary enzyme, EtfD is the only known essential component of *M. tuberculosis* β-oxidation during infection. Interestingly, it has been recently reported that EtfD can reduce menaquinone [116], providing a direct link between β-oxidation and respiration, as observed in humans [117]. EtfD inhibitors were identified, namely the 6,11-dioxobenzo [f]pyrido [1,2-a]indoles (DBPI) [118]; however, their action seems to involve more targets than EtfD [116].

The combination of two compounds to inhibit a functionally redundant process has the drawback of increasing the number of drugs necessary to produce an effect and increase the chances of drug-resistant strains emergence. In turn, if the combination results in a rapid bactericidal effect, such a strategy might prove to be a useful option for TB treatment. In this context, the cytochrome *bc1*-*aa3* oxidase inhibitor Q203 (Telacebec) is advancing in clinical trials with promising data [119,120]; however, the non-essentiality of its main target [75,106,107] makes this drug likely to be bacteriostatic. This Q203 characteristic prompted researchers to exploit the synthetic lethality of *M. tuberculosis* terminal oxidases. The discovery of the cytochrome *bd* oxidase inhibitor ND-011992 showed that the combination of inhibitors to both terminal oxidases rapidly kill *M. tuberculosis* in a mouse model of infection [108], attesting the validity of a drug combination strategy against functional redundancy. The synthetic lethality of NADH dehydrogenase could also be exploited to eradicate *M. tuberculosis* infections in a similar manner [75,97]. Several Ndh-2 inhibitors have already been identified(reviewed in [121]), such as the TriSLas compounds [122]; however, there are no *M. tuberculosis* Ndh-1-specific inhibitors. Most of Ndh-1 inhibitors are active against homologs throughout the tree of life and are extremely toxic, including to humans. Although challenging due to the homology to human complex I, it may be worthwhile developing inhibitors with increased affinity for the *M. tuberculosis* enzyme.

A third strategy to target functional redundancies is to develop inhibitors that target multiple enzymes. In a recent report, a compound (BB2-50F) that simultaneously inhibits succinate dehydrogenases and ATP synthase was shown to kill both replicating and non-replicating *M. tuberculosis* [123]. Although not an example of a drug targeting only a functional redundancy, it shows that targeting phylogenetically unrelated enzymes in a highly redundant process like oxidative phosphorylation is a promising strategy. Multi-enzyme targeting drugs can also be developed to inhibit paralog enzymes. For example, efforts have been ensued to identify inhibitors for enzymes that activate FA, namely FACLs and fatty acyl-AMP ligases [124,125].

Among the outlined strategies, targeting essential components of redundant cell processes is undoubtedly the one with more chances to render successful treatment options. In specific, novel ATP synthase inhibitors are in urgent need to face the challenge of growing cases of bedaquiline resistance [126]. The identification of EtfD inhibitors is also a very promising route for TB drug development for the following reasons: i) it is essential for *M. tuberculosis* growth and survival *in vivo* [74], ii) it does not have human homologs, and iii) it is druggable. Finally, menaquinone biosynthesis inhibitors also hold great promise given that the essentiality of this pathway during infection is confirmed. In all cases, either because of significant structural differences to the human homolog (ATP synthase) or the absence of a human homolog (EtfD or menaquinone biosynthesis enzymes), toxic effects on the host are less likely. Directly targeting functional redundancy can be an effective way of killing *M. tuberculosis* since these processes are key for the adaptation to a changing and heterogeneous environment. However, such a strategy comes with the challenge of overcoming compensatory mechanisms, which is a major hurdle for drug development. The lack of validated lead compounds targeting *M. tuberculosis* isocitrate lyase (Icl1/Icl2), despite being identified as a promising target more than twenty years ago [88,89], is a good example of this challenge. Nevertheless, combination of Q203 (Telacebec)/ ND-011992 shows great promise in eradicating *M. tuberculosis* infections, setting an example that argues against the complete dismissal of targeting functional redundancies as drug targets.

## 5. Concluding remarks

The study of redundant processes opens a wide range of relevant and interesting questions, from their evolutionary history and conservation, to discerning mechanisms underlying the compensatory effects and adaptive strategies, to the development of strategies that can target these processes. *M. tuberculosis* has a curious aspect to its biology, which is the conservation of highly redundant processes, contrasting with other obligate human pathogens. In this review, we described and discussed the adaptation of *M. tuberculosis* to heterogeneous host niches as the driving force for the conservation of functional redundancies in its genome. Due to their importance for adaptation to the host, development of inhibitors targeting these cell processes is likely to be effective against *M. tuberculosis*. By uncovering the evolutionary logic and mechanistic basis of functional redundancies in *M. tuberculosis*, we can both deepen our understanding of its remarkable adaptability and unlock new therapeutic opportunities to overcome this formidable pathogen.
